# Method for a detailed evaluation of respiratory cardiac contributions to blood flow in Fontan circulation

**DOI:** 10.1186/1532-429X-16-S1-P121

**Published:** 2014-01-16

**Authors:** Dominik Daniel Gabbert, Chris Hart, Michael Jerosch-Herold, Inga Voges, Philip Wegner, Eileen Pardun, Traudel Hansen, Heiner Kramer, Carsten Rickers

**Affiliations:** 1 Department of Congenital Heart Disease and Pediatric Cardiology, University Hospital Schleswig-Holstein, Kiel, Schleswig-Holstein, Germany; 2 Harvard Medical School, Women's Hospital, Boston, Schleswig-Holstein, Germany

## Background

A recent MRI pilot study [1] indicated that hemodynamics in the Fontan circulation are mainly dependent on respiration according to flow amplitudes and suggested using a respiratory-triggered 4D PC MRI instead of a ECG-triggered 4D PC MRI. The detailed validation of this conclusion with double-triggered PC on acquisition level requires an extensive development of the pulse sequence. In this study, we outline techniques to justify and derive the recent findings in detail on the stage of post-processing using Realtime PC measurements. The regular respiratory-triggered cine measurement is not decoupled but involves the average cardiac effect as offset. The proposed method allows a complete separation of flow into its components stemming from respiration and ventricular function and it allows to study correlation effects.

## Methods

Three children with hypoplastic left heart syndrome were evaluated after surgical completion of the Fontan circulation (TCPC with lateral intraatrial tunnel). In each patient, a 2D Realtime PC measurement with an acquisition time of 38 ms per image was acquired in the inferior vena cava (IVC). Using the physiology data on ECG and respiration, each image of the realtime MRI is assigned to its phase in the cardiac cycle and in the respiratory cycle and allows to determine a twofold triggered flow-matrix with 40 respiratory phases and 10 ECG phases. Conventional cine distributions are then derived for a single trigger by averaging over the other component. The flow reached the end of the resting expiratory position is attributed to the cardiac function offset and allows for a complete separation of the two effects.

## Results

The proposed method was applied successfully to the IVC in the three children with completed TCPC. In none of the studied cases, a significant correlation between the flow due to respiration cardiac function was found. A single triggering to each of these physiological parameters is therefore justified. A separation of flow into respiratory and cardiac components was performed. The observed flow amplitudes in the respiratory distributions were found to be larger than those in the ECG distributions in agreement to previous findings.

## Conclusions

The method of determining a twofold triggered flow matrix from realtime measurements on the post-processing stage turned out to be a powerful tool for studying correlation effects and decoupling respiratory and cardiac components of the measured flow. Therefore, we aim to increase the number of patients in this study for future statistic evaluation of the results.
